# Prevalence of Depression in the Community from 30 Countries between 1994 and 2014

**DOI:** 10.1038/s41598-018-21243-x

**Published:** 2018-02-12

**Authors:** Grace Y. Lim, Wilson W. Tam, Yanxia Lu, Cyrus S. Ho, Melvyn W. Zhang, Roger C. Ho

**Affiliations:** 1 Monash University, Melbourne, Australia; 2 Alice Lee Centre for Nursing Studies, Yong Loo Lin School of Medicine, National University of Singapore, Singapore, Singapore; 3 Department of Clinical Psychology and Psychiatry/School of Public Health, Zhejiang University College of Medicine, Hangzhou, China; 4 Department of Psychological Medicine, National University Hospital, Singapore, Singapore; 5 National Addiction Management Service, Institute of Mental Health, Singapore, Singapore

**Keywords:** Depression, Risk factors

## Abstract

The prevalence of depression may be affected by changes in psychiatric practices and the availability of online mental health information in the past two decades. This study aimed to evaluate the aggregate prevalence of depression in communities from different countries between 1994 and 2014 and to explore the variations in prevalence stratified by geographical, methodological and socio-economic factors. A total of 90 studies were identified and met the inclusion criteria (n = 1,112,573 adults) with 68 studies on single point prevalence, 9 studies on one-year prevalence, and 13 studies on lifetime prevalence of depression. A random-effects model meta-analysis that was performed to calculate the aggregate point, one-year and lifetime prevalence of depression calculated prevalences of 12.9%, 7.2% and 10.8% respectively. Point prevalence of depression was significantly higher in women (14.4%), countries with a medium human development index (HDI) (29.2%), studies published from 2004 to 2014 (15.4%) and when using self-reporting instruments (17.3%) to assess depression. Heterogeneity was identified by meta-regression and subgroup analysis, and response rate, percentage of women and year of publication, respectively, were determined contribute to depression prevalence. This meta-analysis allows benchmarking of the prevalence of depression during the era when online health information emerged, facilitating future comparisons.

## Introduction

Depression is the most common mental health condition in the general population^1,2^, characterised by sadness, loss of interest or pleasure, feelings of guilt or low self-worth, disturbed sleep or appetite, feelings of tiredness, and poor concentration^3^. In its most severe form, depression can lead to suicide^4,5^ and increased risk of mortality^6^. Depression often runs a chronic course and substantially impairs an individual’s occupational potential^7^ and quality of life^8,9^. The World Health Organisation (WHO) predicted that by the year 2020, depression will rank second in global disease burdens and one of the priority conditions covered by the WHO’s Mental Health Gap Action Programme^10^.

Depression can be reliably diagnosed and treated by primary health physicians in the community^11,12^. To reduce mental health disparities globally, it is important to evaluate the prevalence of depression in the community and variation in prevalence by geographical region, country income, and assessment method^13,14^. Bromet *et al*.^15^ studied 89037 people from 18 countries and concluded that the average lifetime and 12-month prevalence estimates of major depression were 14.6% and 5.5% in high-income countries and 11.1% and 5.9% in low- to middle-income countries. In the past two decades, the emergence of the Internet has provided a new platform to enable the public to search information on depression and to influence health seeking behaviour^16–18^. Electronic health (E-health) allows health resources and health care status to be communicated and transferred by electronic medium^19^. Smartphone applications allow global access, enabling assessment or intervention for adults with depression and other psychiatric disorders^20,21^. Patients report high levels of satisfaction with the E-mental health programme as a self-help tool because it overcomes multiple barriers, including cost, timeliness and concerns regarding confidentiality^22^. Mental health professionals can develop their own smartphone applications to engage their patients^23^. E-health can deliver healthcare remotely to rural populations^24^ and enable patients to have access to the latest treatment guidelines^25^. Online health seeking was more likely among women having a child and experiencing psychological distress, especially those suffering from postnatal depression^26^. The Internet has increased the frequency of self-diagnosis^27^ which may increase the prevalence of self-reported depression, especially for young adults^28^.

As a result, it is crucial to benchmark the prevalence of depression during this period for past and future comparisons. As there are an estimated 350 million people of all ages that suffer from depression worldwide^3,29^, the present meta-analysis aims to establish an aggregate of point prevalence, one-year prevalence, and lifetime prevalence of depression from the larger community pool sample from 1994 to 2014. In this meta-analysis, we aimed to identify moderators accounting for the potential heterogeneity of aggregate prevalence of depression. The point prevalence of depressive symptoms in women is known to be twice as high as in men^30^. Furthermore, there are gender differences in the prediction of lethality in suicide attempts among people suffering from severe depression^31^. A previous study showed that there was response bias when using self-reporting instruments to assess for depression^32^, and the response rate might contribute to heterogeneity. As this meta-analysis covered the period of emergence of Internet access, we explored the effect of the year of publication on the heterogeneity and performed a subgroup analysis based on the year of publication. We hypothesised that the year of publication, female gender and response rate were significant moderators accounting for potential heterogeneity of the aggregate prevalence of depression.

## Results

### Study identification

**Figure 1:**
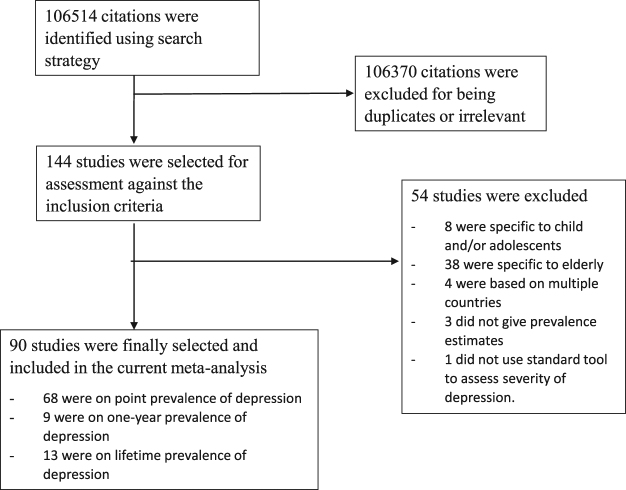
Review and meta-analysis flow diagram.

A total of 106,514 studies were identified after an initial search. After removal of duplicates, we reviewed 144 studies in full. Eight studies were specific to children and/or adolescents, 38 studies were specific to elderly, 4 studies were based on multiple countries and 3 studies did not provide prevalence estimates. After exclusion of the ineligible studies, 91 studies published between April 1994 and June 2014 were finally included. The flow diagram of the search process is shown in Fig. 1. The sample sizes of the reviewed studies ranged from 156 to 235,067 (median 1817), with a total of 1,112,573 participants worldwide.

### Characteristics of the included studies

The prevalence of depression and characteristics of the selected studies are presented in Supplementary Tables 1, 2, and 3. There were 68 (75.56%) studies reporting point prevalence of depression (n = 897,061), 9 (11.11%) studies reporting one-year prevalence of depression (n = 52,163), and 14 (14.44%) studies reporting lifetime prevalence of depression (n = 163,349). For point prevalence of depression, 4 studies were conducted in Africa (n = 3835), 25 studies were conducted in Asia (n = 487,440), 5 studies were conducted in Australia (n = 24,283), 19 studies were conducted in Europe (n = 79,503), 11 studies were conducted in North America (n = 295,279), and 4 studies were conducted in South America (n = 6,721). For the Human Development Index (HDI), there were 35 studies conducted in countries with very high HDI (n = 639,124), 24 studies conducted in countries with high HDI (n = 227,210), 5 studies conducted in countries with medium HDI (n = 26,892), and 4 studies conducted in countries with low HDI (n = 3,835). In terms of the assessment methods, 24 studies utilised an interview-based method (n = 127,082), 43 studies utilised self-report instruments (n = 593,544), and 1 study applied both interview-based and self-report methods (n = 176,435). Eleven studies were conducted in rural settings (n = 9,341), 28 studies were conducted in urban settings (n = 83,890), and 29 studies were conducted in both urban and rural settings (n = 803,830). Twenty-seven studies were conducted in English-speaking countries (n = 349,865), while 41 studies were conducted in non-English-speaking countries (n = 547,194).

### Aggregate prevalence of depression

#### Aggregate point prevalence of depression

**Figure 2:**
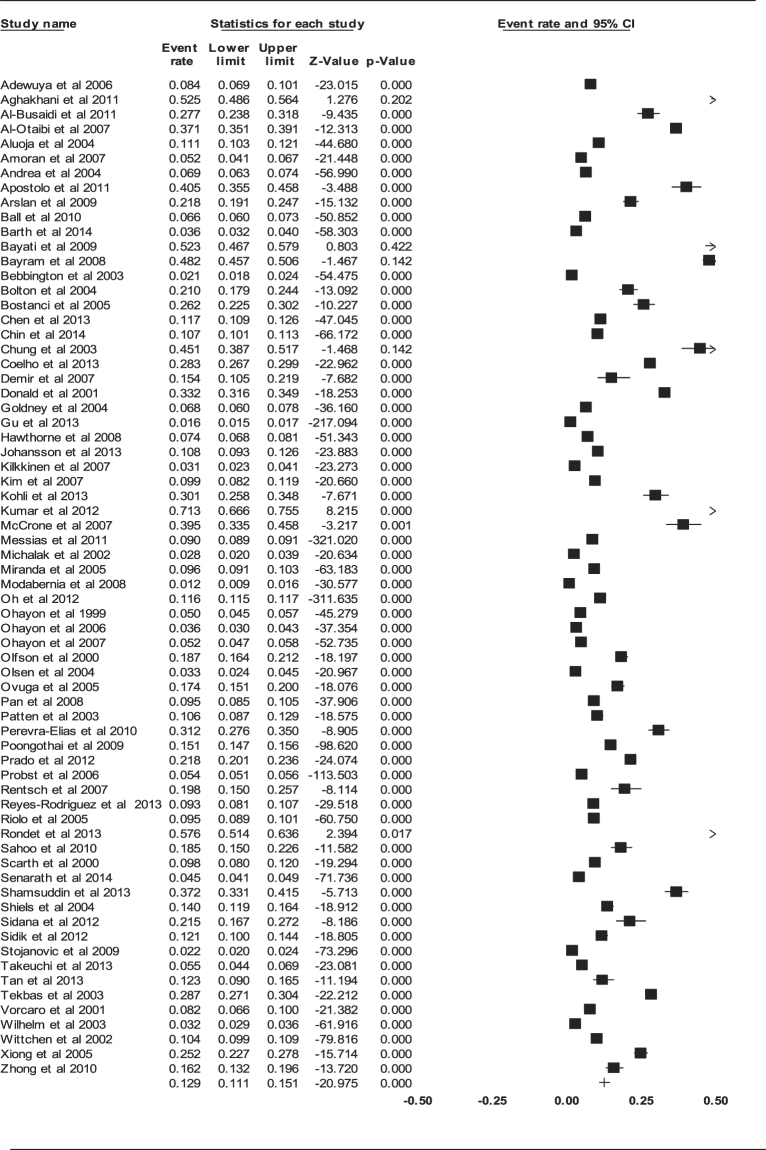
Forest plot of point prevalence of depression.

The aggregate point prevalence of depression of the 68 studies using the random-effect model was 12.9% (95% CI: 11.1–15.1%, *Q* value = 28478.392, *df* = 67, tau^2^ = 0.552) (Fig. 2). There was a significant, high-level of heterogeneity between the studies (*I* ^2^ = 99.765, *P* < 0.001).

#### Aggregate one-year prevalence of depression

**Figure 3:**
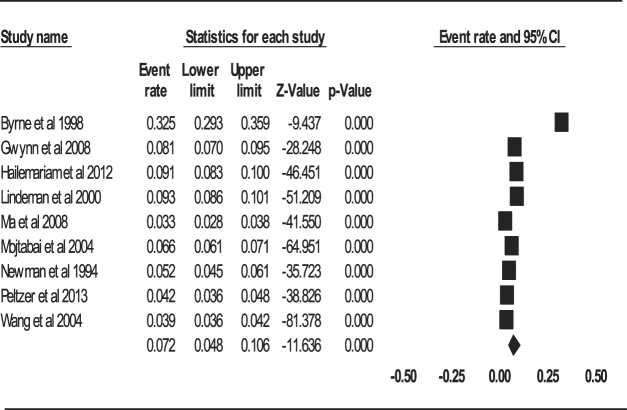
Forest plot of one-year prevalence of depression.

The aggregate one-year prevalence of depression of the 9 studies was 7.2% (95% CI: 4.8–10.6%, *Q* value = 1068.652, *df* = 8, tau^2^ = 0.430) (Fig. 3). There was a significant and high-level of heterogeneity between the studies (*I* ^2^ = 99.251, *P* < 0.001).

#### Aggregate lifetime prevalence of depression

**Figure 4:**
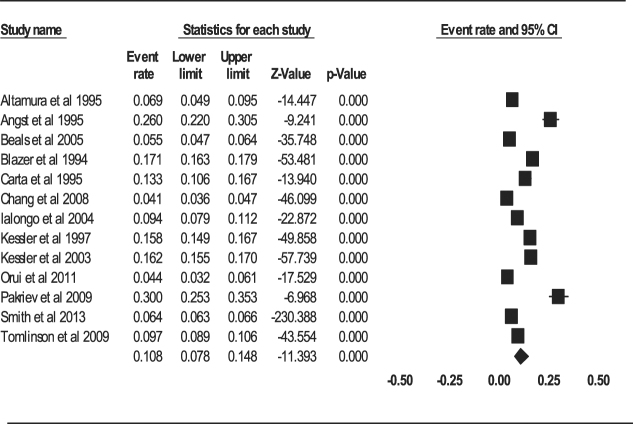
Forest plot of lifetime prevalence of depression.

The aggregate lifetime prevalence of depression with the 13 studies using the random-effect model was 10.8% (95% CI: 7.8–14.8%, *Q* value = 2991.054, *df* = 12, tau^2^ = 0.437) (Fig. 4). There was a significant and high-level of heterogeneity among the studies (*I* ^2^ = 99.599, *P* < 0.001).

### Meta-regression and publication bias

#### Moderators for point prevalence of depression

The response rate (B = 0.0200, z = 2.84, *P* = 0.0044) was a significant moderator that contributed to heterogeneity (Table 1). However, the proportion of women in the study population (B = −0.1507, z = −0.26, *P* = 0.7965) and the year of publication (B = 0.0539, z = 1.73, *P* = 0.0842) were non-significant moderators. There was no evidence of publication bias (intercept = 3.74, 95% CI: −2.62–10.11, t = 1.18, *df* = 66, *P* = 0.244).

**Table 1:** Results for meta-regression on studies on point prevalence of depression.

Predictor	No. of studies used	Univariate coefficient	*Z* value	*P* value	Estimated tau^2^
Percentage of women in study population	58	−0.1507	−0.26	0.7965	0.6776
Year of publication	68	0.0539	1.73	0.0842	0.6590
Response rate (%)	56	0.0200	2.85	0.0044	0.6964

#### Moderators for one-year prevalence of depression

The proportion of women in the study population (B = 4.51, z = 4.38, *P* < 0.001) and the response rate (B = −0.0248, z = −0.0458, *P* = 0.0207) were significant moderators and contributed to heterogeneity (Table 2). The year of publication (B = −0.0446, z = −1.18, *P* = 0.2361) was not a significant moderator. There was no evidence of publication bias (intercept = 6.19, 95% CI: −24.90–37.38, t = 0.471, *df* = 7, *P* = 0.652).

**Table 2:** Results for meta-regression on studies on one-year prevalence of depression.

Predictor	No. of studies used	Univariate coefficient	*Z* value	*P* value	Estimated tau^2^
Percentage of women in study population	9	4.5144	4.38	<0.001	0.1876
Year of publication	9	−0.0446	−1.18	0.2361	0.4572
Response rate (%)	9	−0.0248	−0.0458	0.0207	0.3199

#### Moderators for lifetime prevalence of depression

The year of publication (B = −0.0487, z = −2.773, *P* = 0.00556) was a significant moderator and contributed to heterogeneity. The proportion of women in the study population (B = −3.141, z = −20.463, *P* = 0.643) and the response rate (B = 0.0249, z = 1.471, *P* = 0.141) were non-significant as moderators (Table 3). There was no evidence of publication bias (intercept = 7.95424, 95% CI: −5.295–21.203, t = 1.321, *df* = 11, *P* = 0.213).

**Table 3:** Results for meta-regression on studies on lifetime prevalence of depression.

Predictor	No. of studies used	Univariate coefficient	*Z* value	*P* value	Estimated tau^2^
Percentage of women in study population	12	−15.1026	−2.37	0.0178	0.5898
Year of publication	14	−0.0341	−1.34	0.1788	0.3677
Response rate (%)	11	0.0443	2.80	0.0051	0.3819

### Subgroup analysis

Subgroup analysis was performed for studies reporting point prevalence of depression (Table 4).

**Table 4:** Point prevalence of depression according to different category.

Category	Subgroup	No. of studies	Pooled Prevalence (95% CI) (%)	*N*	*P* value in between-group comparison
Continent	Africa	4	11.5 [6.3–20.1]	3835	
	Asia	25	16.7 [13.5–20.4]	487440	
	Australia	5	7.3 [2.4–20.2]	24283	
	Europe	19	11.9 [7.5–18.4]	79503	
	North America	11	13.4 [10.6–16.9]	295279	
	South America	4	20.6 [13.8–29.7]	6721	
	Overall:	68			0.158
Human development index	Very High	35	9.8 [8.4–11.3]	639124	
	High	24	19.2 [13.9–26.0]	227210	
	Medium	5	29.2 [13.5–52.0]	26892	
	Low	4	11.5 [6.3–20.1]	3835	
	Overall:	64			<0.001
Assessment method	Interview-based	24	8.5 [6.5–11.0]	127082	
	Self-report	43	17.3 [15.0–19.9]	593544	
	Overall:	67			<0.001
Setting	Rural	11	13.0 [8.5–19.3]	9341	
	Urban	28	17.7 [13.0–23.6]	83890	
	Overall:	39			0.228
Language of the countries	English speaking countries	27	14.2 [10.8–18.5]	349865	
	Non-English speaking countries	41	13.4 [11.5–15.6]	547196	
	Overall	68			0.710

#### Continent

Among continents, South America had the highest aggregate prevalence at 20.6% (95% CI: 13.8–29.7%). This was followed by Asia at 16.7% (95% CI: 13.5–20.4%), North America at 13.4% (95% CI: 10.6–16.9%), Europe at 11.9% (95% CI: 7.5–18.4%), and Africa at 11.5% (95% CI: 6.3–20.1%). Australia had the lowest prevalence of depression at 7.3% (95% CI: 2.4–20.2%). The differences were not significant (*P* = 0.158).

#### Human development index (HDI)

Regarding the HDI, the aggregate prevalence was 9.8% (95% CI: 8.4–11.3%) for very high HDI, 19.2% (95% CI: 13.9–26.0%) for high HDI, 29.2% (95% CI: 13.5–52.0%) for medium HDI, and 11.5% (95% CI: 6.3–20.1%) for low HDI. The differences were significant (*P* < 0.001).

#### Assessment method

The prevalence for studies using self-report instruments was higher (17.3%, 95% CI: 15.0–19.9%) than that of studies using interview-based assessment tools (8.5%, 95% CI: 6.5–11.0%). The difference was significant (*P* < 0.01).

#### Gender

Regarding gender differences, the aggregate prevalence was 14.4% (95% CI: 11.1% to 11.7%) for women and 11.5% (95% CI: 9% to 14.6%) for men. The difference was significant (*P* < 0.001).

#### Year of publication

Regarding the year of publication, the aggregate prevalence was 15.4% (95% CI: 12.9% to 18.3%) for studies published from 2004 to 2014 and 9.8% (95% CI: 6.7% to 14.1%) for studies published from 1994 to 2003. The difference was significant (*P* < 0.001).

#### Setting

The aggregate prevalence for studies conducted in rural settings was 13.0% (95% CI: 8.5–19.3%). This prevalence was lower than the prevalence of studies conducted in urban settings (17.7%, 95% CI: 13.0–23.6%). The difference was not significant (*P* = 0.228).

#### Language

Studies conducted in English-speaking countries had a higher aggregate prevalence of 14.2% (95% CI: 10.8–18.5%) compared to studies conducted in non-English speaking countries. The aggregate prevalence for non-English speaking countries was 13.4% (95% CI: 11.5–15.6%). The difference was not significant (*P* = 0.710).

## Discussion

This meta-analysis aimed to provide an up-to-date estimate of the prevalence of depression among adults in the community by combining the data of 1 million participants from 30 countries from 1994 to 2014, which marked the era of broad access to the Internet, E-health and online access to health information. We observed that the aggregate point prevalence was 12.9%, one-year prevalence was 7.2%, and lifetime prevalence was 10.8% from 1994 to 2014.

There are a number of reasons that might explain the paradoxically higher point prevalence of depression compared to lifetime prevalence of depression. Recall bias^33^ may have led people to under-report past depressive episodes, resulting in a lower prevalence in studies that reported on lifetime depression. Similarly, people who suffered from depression might have periods of remission and might not report depressive symptoms during their remission periods. Additionally, compared to past decades, depression is better accepted as a medical condition by both patients and health professionals. Even if patients did experience depressive episodes in the past, they might not be given a formal diagnosis, thereby accounting for the lower prevalence in studies that reported lifetime depression.

Our findings suggest that depression is a common and substantial mental health problem in the community worldwide in the past two decades during the era of emergence of Internet and online health information. A wide range of populations was examined and contributed to the significant heterogeneity in prevalence across studies. The meta-regression identified such factors as proportion of women in the population, response rate in the survey and year of publication as contributing to the heterogeneity in the aggregate prevalence of depression.

Prevalence of depression appeared to be significantly higher as measured by self-report instruments versus clinical interviews (17.3% vs 8.5%). According to the WHO, an important barrier to effective care for depression is inaccurate assessment and that people who are depressed are often not correctly diagnosed^10,34^. In this study, the aggregate prevalence of depression was significantly higher in self-reported instruments compared to diagnostic interviews. Our findings correspond to a previous meta-analysis^35,36^. Studies that utilised self-rated depression scales produced a significantly higher point prevalence of depression compared to studies using clinician-rating scales. Rush *et al*. established that for milder forms of depression, more depressive symptoms were elicited through self-reported scales^37^. The discrepancies between self-report and clinician-rating instruments could be related to patient personality and demographic factors. Patients who scored higher on self-report instruments were younger, more educated, and had higher neuroticism, lower extraversion, and lower agreeableness^38^. Conversely, clinician-rating scales were shown to be more effective in evaluating patients of advanced age, less education, and with psychotic features and limited insight^39^. Furthermore, diagnostic interviews adopt a more stringent criteria and identify those with a depressive disorder but not with depressive symptoms^36^. This may lead to unmet mental health needs for people suffering from depressive symptoms in the community. This indicates that there is under-detection if diagnostic interviews are used alone, which may lead to a delay in treatment of depressive symptoms during the early stage of the illness. Diagnostic interviews are time-consuming and labour intensive^36^ and this may hamper routine implementation of screening for depression in the community setting. Given the strengths and limitations of each of the assessment methods, we recommend the use of a multi-modal assessment approach that involves both self-reporting (assessment) and a shortened version of a diagnostic interview (case-finding) to establish the prevalence of depression for future epidemiological studies.

Our study has several implications for service development. First, point prevalence of depression was highest in countries with a medium HDI. Our finding is different from previous studies and suggests a changing trend in the past decades. It posits that people living in medium HDI countries may be exposed to more stressors due to higher expectations and costs of living, as well as a higher cost of managing depression^40^ than low HDI countries. These factors, combined with greater stigma and social inequalities, may lead to a lesser likelihood of receiving or seeking treatment for depression. Our data suggest that developing an equitable community-based mental health programme may be beneficial to reduce the prevalence of depression in medium HDI countries. The World Health Survey (WHS) conducted in 2013 showed that the prevalence of depression for countries classified according to economic development was similar, with 6.0% in low-income countries, and 7.6% in upper-middle-income countries^41^. An earlier study conducted in 2008 based on the WHS results found that countries with a low HDI had the highest one-year prevalence of depression at 6.5%, and countries with middle HDI had the lowest one-year prevalence at 3.9%^42^. It should be noted that these two studies focussed on the one-year prevalence of depression, while our analysis was based on the point prevalence of depression.

This meta-analysis did not find a difference in point prevalence of depression between urban and rural settings. Similarly, a systematic review by Judd *et al*.^43^ failed to demonstrate a significant difference in the prevalence of depression between urban and rural settings, and suggested that socio-demographic factors common in both urban and rural settings may be more predictive of depression. This is supported by findings of a 2002 Korean study on late-life depression^44^. In the urban sample, advanced age and poor education were found to be independently associated with depression. Similarly, low education was found to be associated with depression in the rural sample. It is possible that people in either rural or urban environments have common characteristics that are strongly associated with depression. Future research should focus on identifying particular groups in both the urban and rural settings whose risk of depression is increased. Moreover, recent advances in technology (e.g., Internet and railway systems) have narrowed the differences between urban and rural areas. People living in rural areas may have access to mental health information and visit cities more frequently and economically. There was no significant difference in the point prevalence of depression between studies conducted in English-speaking and non-English speaking countries, or in the point prevalence between continents. This could be because the differences in point prevalence of depression between countries is better explained by differences in HDI.

The response rate was a significant moderator and was associated with heterogeneity of the point and one-year prevalence of depression. Studies with a high response rate tended to report higher point and one-year prevalence of depression scores compared to studies with low response rates. This finding might be explained by response bias – participants who were willing to take part in the study might be more likely to report depressive symptoms as influenced by how the questions were asked in the self-report questionnaires or structured interviews. It is interesting to note that the response rate was not a significant moderator for life-time prevalence depression. There are several explanations for this finding. First, the depressive episode could have occurred long time ago which did not affect the potential participants during the time of study recruitment, regardless of whether they agreed or disagreed to participate in the study. Second, participants with depression might suffer from cognitive impairment which could affect their ability to recall past depressive episodes^45^. The proportion of women in the study population was a significant moderator accounting for the heterogeneity in the one-year prevalence of depression. Our findings support earlier findings that the prevalence of depression is higher among women^46,47^ and there is still a gender effect on the prevalence of depression^35,48^. In the past two decades, the social standing of women has improved and the income gap has narrowed between men and women. The prevalence of depression between women and men was reported to be in the ratio of 2: 1^30^. In this study, the subgroup analysis showed that the female to male ratio was approximately 1.25: 1, which might explain the lack of gender effect on the point and lifetime prevalence of depression in this meta-analysis. Furthermore, the gender difference in one-year prevalence of depression could have been narrowed. The year of publication was found to be a significant moderator for the lifetime prevalence of depression, and the coefficient was in the negative direction. This finding implies that heterogeneity was greater in the earlier studies, but it has decreased in more recent studies, which could be due to an advancement of E-health where participants have an equal opportunity to access health information from the Internet. Although the year of publication was not a significant moderator to explain heterogeneity for point and one-year prevalence of depression, a subgroup analysis showed that studies published from 2004 to 2014 had a significantly higher prevalence of depression (15.4%) compared to studies published from 1994 to 2003 (9.8%). This finding supports our hypothesis that the development of the Internet and E-health has improved the awareness of depression among participants who participated in recent studies.

The strength of this current study includes a comprehensive meta-analysis of a large number of studies globally using a meta-regression analysis and subgroup analysis. Furthermore, the studies consisted of people from the community; this avoids the selection bias present in the clinical setting. Therefore, the findings are most applicable to family physicians and public health policy makers in formulating strategies to lessen the burden of depression in the community. This meta-analysis includes studies conducted in almost all continents, accounting for cultural and ethnic differences in lifestyle and genetic factors. In addition, this study includes both self-report instruments and diagnostic interviews. It is important to analyse both assessment methods, as both methods are commonly used to assess the severity of depression^36^. Other strengths include a lack of significant publication bias and the utilisation of random-effects models to establish robust aggregate prevalence^49^.

This study has several limitations. First, this meta-analysis has a high level of heterogeneity. Heterogeneity appears to be the norm rather than an exception in meta-analyses of large numbers of studies globally^49^. Second, due to limited data, a meta-regression could not be performed on other moderators such as family history of depression, social status of individuals, marital status, and medical comorbidity. Depression is reportedly associated with a number of specific chronic illnesses^50^. Third, meta-regression represents an observational association and is limited by ecological fallacy^51^ and cross-sectional information cannot identify a temporal causality. Fourth, almost all of the included studies were of cross-sectional design except two prospective cohort studies that were included. More longitudinal studies are required in the future because they can determine the temporal association between predisposing factors and the risk of developing depression. Fifth, subgroup analyses were not performed for one-year and lifetime-prevalence of depression due to the small number of studies available. Despite these limitations, this meta-analysis provides the most up-to-date information on point, one-year, and lifetime prevalence of depression in the community in the past two decades.

In conclusion, this meta-analysis identified that the aggregate point prevalence, one-year, and lifetime prevalence of depression were 12.9%, 7.2%, and 10.8%, respectively from 1994 to 2014, and the heterogeneity in prevalence was high. Women had a higher prevalence of depression but the gender difference could have been narrowed. This meta-analysis allows benchmarking of the prevalence of depression during the era of emerging online health information for past and future comparisons. The subgroup analysis showed that the prevalence of depression in studies published from 2004 to 2014 was higher than those published from 1994 to 2003. The use of diagnostic interviews may underestimate the prevalence of depressive symptoms. Combined self-report instruments and short-form diagnostic interviews are recommended to screen and identify for depressive cases in future epidemiological studies. Meta-regression analyses showed that the response rate, proportion of female gender, and year of publication were determinants of heterogeneity in the prevalence of depression.

## Methods

### Study identification

The databases Embase, MEDLINE, PsycINFO, PubMed, Science Direct, and Web of Science were systemically reviewed to identify studies published between April 1994 and June 2014. Keywords were “depress” OR “depression” OR “depressive” OR “depressive disorder” OR “major depression” AND “preval” OR “prevalence” OR “epidemiol”, “epidemiologic” OR “epidemiological” studies”. Two authors (G.Y.L. and R.C.H.) independently screened the titles and abstracts and reviewed the full-text of the eligible articles. References of eligible articles were also screened for other relevant studies.

### Selection criteria

For a study to be included in this meta-analysis, two authors (G.L. and R.H.) must have agreed that the study met the inclusion criteria. We used studies for this meta-analysis that met the following criteria:

(i) Studies were cross-sectional or cohort by design and were conducted in a single country;
(ii) Studies stated the prevalence of depression or the relative available data (the number of participants with depression and total number of participants) to calculate the prevalence of depression;
(iii) Studies were based on epidemiological surveys in a community;
(iv) Studies assessed depressive symptoms by validated diagnostic or self-report instruments; and
(v) Studies were published in peer-reviewed journals with an English abstract and full-text available.

We excluded the studies that met any of the following criteria:

(i) Studies that did not provide relevant data for the prevalence of depression;
(ii) Studies that were based on special populations (e.g., specific medical or psychiatric conditions or caregivers);
(iii) Studies that were based on children and adolescents (<18 years) or elderly (>65 years); and
(iv) Duplicate studies or studies that were contained within another study.

### Data extraction

Data extraction was in accordance with the Preferred Reporting Item for Systematic Reviews and Meta-Analysis: The PRISMA statement^52^. Two investigators (G.Y.L. and R.C.H.) extracted information from all eligible publications independently. Disagreement was discussed and resolved by a third investigator (Y.L.). The following information was extracted from each study and the data extraction form was filled: Family name of the first author, year of publication, country, setting (rural or urban), age range and mean age, proportion of women in the study population, self-report or clinician rating of depressive symptoms, instruments to assess depressive symptoms, sample size, response rate, number of people suffering depression in the community, total population screened in the community, prevalence estimation, and sex-specific prevalence. The studies were classified into 3 groups according to their study endpoints – point prevalence of depression, one-year prevalence of depression, and lifetime prevalence of depression.

### Statistical analysis

Statistical analyses were completed using the Comprehensive Meta-Analysis Version 2.0 programme. Because of the expected heterogeneity across studies, a random-effects model was used to calculate the aggregate prevalence and 95% confidence intervals (CIs). The random-effect model was used because it assumes varying effect sizes between studies, and because of differing study designs and study populations^53^. Between-study heterogeneity was evaluated with the Cochran chi-square and quantified with the I^2^ statistic, which was used to estimate total variation across studies due to heterogeneity rather than chance. Mixed-effects meta-regression was conducted to identify moderators that might contribute to the heterogeneity or observed variations between studies. The covariates examined were publication year, proportion of women in the study population and the response rate. The regression coefficients, the associated z values and the *P* values were reported in the meta-regression analysis. Publication bias was evaluated using funnel plots and the modified Egger’s linear regression test. Significance was set at *P* < 0.05. For studies reporting point prevalence of depressive disorders, subgroup analysis was performed while the subgroups were divided according to continent (North America, South America, Europe, Africa, Asia, Australia), setting (urban, rural), assessment method (self-rated, interview-based), 2013 HDI (very high, high, medium, low), and language of the countries (English-speaking countries, non-English speaking countries). HDI is a composite statistic of indicators of life expectancy, education, and income per capita, which ranks countries into four tiers. A very high HDI is more than 0.8, a high HDI is between 0.7 and 0.799, a medium HDI is between 0.55 and 0.699, and a low HDI is less than 0.549. Subgroup analysis was not considered for studies reporting on one-year prevalence and lifetime prevalence due to insufficient studies in these two categories.
