# Author Correction: Prevalence of Depression in the Community from 30 Countries between 1994 and 2014

**DOI:** 10.1038/s41598-022-19021-x

**Published:** 2022-09-01

**Authors:** Grace Y. Lim, Wilson W. Tam, Yanxia Lu, Cyrus S. Ho, Melvyn W. Zhang, Roger C. Ho

**Affiliations:** 1 Monash University, Melbourne, Australia; 2 Alice Lee Centre for Nursing Studies, Yong Loo Lin School of Medicine, National University of Singapore, Singapore, Singapore; 3 Department of Clinical Psychology and Psychiatry/School of Public Health, Zhejiang University College of Medicine, Hangzhou, China; 4 Department of Psychological Medicine, National University Hospital, Singapore, Singapore; 5 National Addiction Management Service, Institute of Mental Health, Singapore, Singapore

Correction to: *Scientific Reports* 10.1038/s41598-018-21243-x, published online 12 February 2018

The Supplementary Information file was erroneously omitted from the original version of this Article. The original Supplementary Information file is provided below.

## Supplementary Information

Supplementary Information.

