# Supplementary material for: Author Correction: Prevalence of Depression in the Community from 30 Countries between 1994 and 2014
Source: Sci Rep. 2022 Sep 1;12:14856. doi: 10.1038/s41598-022-19021-x (PMC9436937; doi:10.1038/s41598-022-19021-x)
Supplement: Supplementary file 1 — Supplementary Information. [file 41598_2022_19021_MOESM1_ESM.docx]

**Prevalence of Depression in the Community from 30 Countries between1994 and 2014**

Grace Y Lim,^1^ Wilson W Tam,^2^ Yanxia Lu,^3^ Su Hui Ho,^4^ Melvyn W. Zhang,^5^ Roger C. Ho,^4^

**Supplementary Table 1**  Characteristics of studies on point prevalence of depression

| **No.** | **First author** | **Publication year** | **Age (mean years)** | **Female percent (%)** | **Continent** | **Country** | **Setting** | **Language** | **HDI** | **Assessment method** | **Assessment tool** | **Response rate (%)** | **Event** | **Sample size** | **Prevalence (%)** |
| --- | --- | --- | --- | --- | --- | --- | --- | --- | --- | --- | --- | --- | --- | --- | --- |
| 1 | Adewuya | 2006 | 24.98 | 42.5 | Africa | Nigeria | Urban | English | Low | QI | MINI | 92.8 | 101 | 1206 | 8.4 |
| 2 | Aghakhani | 2011 | 22 | 46.8 | Asia | Iran | Urban | English | High | Q | BDI | 89.7 | 330 | 628 | 52.6 |
| 3 | Al-Busaidi | 2011 | 20.75 | 49.5 | Asia | Oman | Urban | Other | High | Q | PHQ-9 | - | 133 | 481 | 27.7 |
| 4 | Al-Otaibi | 2007 | 21-64 | 53.3 | Asia | Kuwait | Mixed | Other | Very High | Q | BDI-II | 97.8 | 860 | 2320 | 37.1 |
| 5 | Aluoja | 2004 | 15-79 | 54.9 | Europe | Estonia | Mixed | Other | Very High | Q | EST-Q | 99.3 | 521 | 4677 | 11.1 |
| 6 | Amoran | 2007 | 34.34 | 62.4 | Africa | Nigeria | Mixed | English | Low | QI | SCI DSM IV | 90.6 | 58 | 1105 | 5.2 |
| 7 | Andrea | 2004 | 40.9 | 27.4 | Europe | Netherlands | Urban | Other | Very High | Q | HADS | 70 | 512 | 7472 | 6.9 |
| 8 | Apostolo | 2011 | 43.82 | 72.9 | Europe | Portugal | Mixed | Other | Very High | Q | DASS-21 | - | 139 | 343 | 40.5 |
| 9 | Arslan | 2009 | 20.82 | 54.1 | Europe | Turkey | Rural | Other | High | Q | BDI | 80.8 | 179 | 822 | 21.8 |
| 10 | Ball | 2010 | - | 54.0 | Asia | Sri Lanka | Mixed | Other | High | QI | CIDI | 91.7 | 395 | 5973 | 6.6 |
| 11 | Barth | 2014 | 19.7 | 0 | Europe | Switzerland | Mixed | English | Very High | Q | PHQ-9 | - | 326 | 9066 | 3.6 |
| 12 | Bayati | 2009 | 20.9 | 48.0 | Asia | Iran | Urban | Other | High | Q | GHQ-28 | 86.8 | 159 | 304 | 52.3 |
| 13 | Bayram | 2008 | 20.5 | 55.9 | Europe | Turkey | Urban | Other | High | Q | DASS-42 | 100 | 779 | 1617 | 48.2 |
| 14 | Bebbington | 2003 | 16-64 | 50.4 | Europe | UK | Mixed | English | Very High | QI | CIS-R | 100 | 205 | 9792 | 2.1 |
| 15 | Bolton | 2004 | 39.3 | 62.0 | Africa | Uganda | Rural | English | Low | QI | DHSCL | 98 | 123 | 587 | 21.0 |
| 16 | Bostanci | 2005 | 20.4 | 41.3 | Europe | Turkey | Urban | Other | High | Q | BDI | 100 | 132 | 504 | 26.2 |
| 17 | Chen | 2013 | 21.2 | 51.1 | Asia | China | Urban | Other | High | Q | BDI | 91.3 | 614 | 5254 | 11.7 |
| 18 | Chin | 2014 | 49 | 56.6 | Asia | Hong Kong | Mixed | Other | Very High | Q | PHQ-9 | 81 | 1089 | 10179 | 10.7 |
| 19 | Chung | 2003 | 51.08 | 65.2 | North America | USA | Urban | English | Very High | Q | CES-D | 83.4 | 101 | 224 | 45.1 |
| 20 | Coelho | 2013 | 14+ | 57.2 | South America | Brazil | Mixed | Other | High | Q | CES-D | 66.4 | 851 | 3007 | 28.3 |
| 21 | Demir | 2007 | 28.2 | 59.6 | Europe | Turkey | Urban | Other | High | Q | BDI | 75.3 | 24 | 156 | 15.4 |
| 22 | Donald | 2001 | 18.55 | - | Australia | Australia | Urban | English | Very High | Q | CES-D | 67.3 | 1024 | 3082 | 33.2 |
| 23 | Goldney | 2004 | - | - | Australia | Australia | Mixed | English | Very High | QI | PRIME-MD | 70.2 | 205 | 3010 | 6.8 |
| 24 | Gu | 2013 | - | - | Asia | China | Mixed | Other | High | Q, QI | GHQ-12, CIDI, ICD-10 | - | 2823 | 176435 | 1.6 |
| 25 | Hawthorne | 2008 | 15+ | 51.1 | Australia | Australia | Mixed | English | Very High | QI | PRIME-MD | 67 | 446 | 6025 | 7.4 |
| 26 | Johansson | 2013 | 46.2 | 56.1 | Europe | Sweden | Mixed | Other | Very High | Q | PHQ-9 | 44.3 | 144 | 1329 | 10.8 |
| 27 | Kilkkinen | 2007 | 25-74 | 53.5 | Australia | Australia | Rural | English | Very High | Q | HADS | 45.9 | 47 | 1525 | 3.1 |
| 28 | Kim | 2007 | 20+ | 57.9 | Asia | South Korea | Urban | Other | Very High | Q | CES-D | 93.4 | 97 | 981 | 9.9 |
| 29 | Kohli | 2013 | 31.73 | 67.1 | Asia | India | Rural | Other | Medium | Q | PHQ-9 | - | 119 | 395 | 30.1 |
| 30 | Kumar | 2012 | - | 45.8 | Asia | India | Urban | Other | Medium | Q | BDI | - | 285 | 400 | 71.3 |
| 31 | McCrone | 2007 | 35 | 50.8 | North America | USA | Rural | English | Very High | Q | BDI | 88.1 | 94 | 238 | 39.5 |
| 32 | Messias | 2011 | - | - | North America | USA | Mixed | English | Very High | Q | PHQ-9 | 52.0 | 21156 | 235067 | 9.0 |
| 33 | Michalak | 2002 | 18-64 | - | Europe | UK | Rural | English | Very High | Q | BDI, SCAN | - | 35 | 1239 | 5.1 |
| 34 | Miranda | 2005 | 30.73 | 100 | North America | USA | Urban | English | Very High | QI | PRIME-MD | 56.2 | 882 | 9151 | 9.6 |
| 35 | Modabernia | 2008 | 18-70 | 62.7 | Asia | Iran | Mixed | Other | High | Q | BDI | 13 | 49 | 4020 | 1.2 |
| 36 | Oh | 2012 | 49.5 | 53.6 | Asia | South Korea | Mixed | Other | Very High | Q | CES-D | - | 26619 | 229595 | 11.6 |
| 37 | Ohayon | 1999 | 15+ | - | Europe | UK | Mixed | English | Very High | QI | DSM-IV | 79.6 | 250 | 4972 | 5.0 |
| 38 | Ohayon | 2006 | 15-90 | 50.5 | Asia | South Korea | Mixed | Other | Very High | QI | DSM-IV | 86.1 | 134 | 3719 | 3.6 |
| 39 | Ohayon | 2007 | 18-96 | 51.8 | North America | USA | Mixed | English | Very High | QI | DSM-IV | 83.3 | 348 | 6694 | 5.2 |
| 40 | Olfson | 2000 | 53.20 | 75.0 | North America | USA | Urban | English | Very High | QI | PRIME-MD | 79.5 | 188 | 1007 | 18.9 |
| 41 | Olsen | 2004 | 20-79 | 54.0 | Europe | Denmark | Mixed | Other | Very High | Q | MDI | 60.2 | 40 | 1205 | 3.3 |
| 42 | Ovuga | 2005 | 33.1 | 33.1 | Africa | Uganda | Rural | Other | Low | Q | BDI-SF | 93.8 | 163 | 937 | 17.4 |
| 43 | Pan | 2008 | 58.6 | 55.7 | Asia | China | Mixed | Other | High | Q | CES-D | 93.1 | 312 | 3289 | 9.5 |
| 44 | Patten | 2003 | - | 65.7 | North America | Canada | Rural | English | Very High | QI | CIDI-SF | 71.1 | 85 | 801 | 10.6 |
| 45 | Perevra-Elias | 2010 | 18.97 | 71.0 | South America | Peru | Urban | Other | High | Q | Brief Zung scale | 68 | 184 | 590 | 31.2 |
| 46 | Poongothai | 2009 | 39 | 50.6 | Asia | India | Urban | Other | Medium | Q | PHQ-12 | 97.9 | 3847 | 25455 | 15.1 |
| 47 | Prado | 2012 | - | 58.3 | South America | Brazil | Urban | Other | High | QI | CIDI-SF | 74.9 | 454 | 2083 | 21.8 |
| 48 | Probst | 2006 | 18+ | 52.1 | North America | USA | Mixed | English | Very High | QI | CIDI-SF | 100 | 1653 | 30801 | 5.4 |
| 49 | Rentsch | 2007 | 49.5 | 38.2 | Europe | Switzerland | Urban | Other | Very High | Q | PHQ-9 | 66.7 | 42 | 212 | 19.8 |
| 50 | Reyes-Rodriguez | 2013 | 18.26 | 67.8 | North America | Puerto Rico | Urban | English | High | Q | BDI | - | 186 | 1992 | 9.3 |
| 51 | Riolo | 2005 | 15-40 | - | North America | USA | Mixed | English | Very High | QI | DIS | 96.1 | 803 | 8449 | 9.5 |
| 52 | Rondet | 2013 | 45 | 48.4 | Europe | France | Urban | Other | Very High | QI | MINI | 98 | 144 | 250 | 56.7 |
| 53 | Sahoo | 2010 | 19.5 | 0 | Asia | India | Urban | Other | Medium | QI | MINI | 81 | 75 | 405 | 18.5 |
| 54 | Scarth | 2000 | 50.12 | 0 | North America | USA | Rural | English | Very High | Q | CES-D | 58 | 84 | 855 | 9.8 |
| 55 | Senarath | 2014 | 43.2 | 56.6 | Asia | Sri Lanka | Mixed | Other | High | Q | PHQ-9 | 98.8 | 576 | 12841 | 4.5 |
| 56 | Shamsuddin | 2013 | 20.8 | 55.3 | Asia | Malaysia | Urban | English | High | Q | DASS-21 | 91.7 | 188 | 506 | 37.2 |
| 57 | Shiels | 2004 | 44 | 0 | Europe | UK | Rural | English | Very High | Q | HADS | 91.8 | 126 | 901 | 14.0 |
| 58 | Sidana | 2012 | - | - | Asia | India | Urban | Other | Medium | Q | PHQ-9 | 79 | 51 | 237 | 21.5 |
| 59 | Sidk | 2012 | 30.9 | 100 | Asia | Malaysia | Urban | Other | High | Q | PHQ-9 | 87.5 | 102 | 845 | 12.1 |
| 60 | Stojanovic | 2009 | - | - | Europe | Croatia | Urban | Other | Very High | QI | ICD-10 | - | 383 | 17290 | 2.2 |
| 61 | Takeuchi | 2013 | 20-69 | 13.1 | Asia | Japan | Urban | Other | Very High | QI | SCID-CV | 96.3 | 70 | 1266 | 5.5 |
| 62 | Tan | 2013 | 35.9 | 59.5 | Asia | Malaysia | Urban | English | High | Q | PHQ-9 | - | 37 | 301 | 12.3 |
| 63 | Tekbas | 2003 | 20.7 | 0 | Europe | Turkey | Mixed | Other | High | Q | PHQ-9 | 74.4 | 835 | 2910 | 28.7 |
| 64 | Vorcaro | 2001 | 18+ | 56.6 | South America | Brazil | Rural | Other | High | QI | CIDI | 85.3 | 85 | 1041 | 8.2 |
| 65 | Wilhelm | 2003 | 18-75 | - | Australia | Australia | Mixed | English | Very High | QI | CIDI | 78.1 | 341 | 10641 | 3.2 |
| 66 | Wittchen | 2002 | - | 41.0 | Europe | Germany | Mixed | Other | Very High | Q | ICD-10 | 87.8 | 1537 | 14746 | 10.4 |
| 67 | Xiong | 2005 | 19.93 | 0 | Asia | China | Mixed | Other | High | Q | SDS | 90.7 | 279 | 1107 | 25.2 |
| 68 | Zhong | 2010 | 18+ | 52.8 | Asia | China | Mixed | Other | High | QI | SCID-1 | - | 83 | 513 | 16.2 |

MINI: Mini International Neuropsychiatric Interview; BDI: Beck Depression Inventory; PHQ: Patient Health Questionnaire; CIDI: Composite International Diagnostic Interview; EST-Q: Emotional state questionnaire; SCI DSM IV: Structured Clinical Interview DSM IV; HADS: Hospital Anxiety and Depression Scale; SPIKE: Structured Psychopathological Interview and Rating of the Social Consequences of Psychological Disturbances for Epidemiology; DASS-21: Depression Anxiety Stress Scales 21; GHQ-28: General Health Questionnaire; CIS-R: Clinical Interview Schedule-Revised; CES-D: Center for Epidemiologic Studies Depression Scale; PRIME-MD: Primary Care Evaluation of Mental Disorders screening questionnaire for depressive symptoms; SCAN: Schedules for Clinical Assessment in Neuropsychiatry; DIS: Diagnostic Interview Schedule; MDI: Major Depression Inventory; SCID-CV: Structured Clinical Interview for DSM Disorders; SDS: Self-rating Depression Scale.

**Supplementary Table 2**  Characteristics of studies on one-year prevalence of depression

| **No.** | **First author** | **Publication year** | **Age (mean years)** | **Female percent (%)** | **Continent** | **Country** | **Setting** | **Language** | **HDI** | **Assessment method** | **Assessment tool** | **Response rate (%)** | **Event** | **Sample size** | **Prevalence (%)** |
| --- | --- | --- | --- | --- | --- | --- | --- | --- | --- | --- | --- | --- | --- | --- | --- |
| 1 | Byrne | 1998 | 32.4 | 96.7 | North America | Canada | Mixed | English | Very High | QI | CIDI-UM | 44.5 | 247 | 760 | 32.5 |
| 2 | Gwynn | 2008 | 20+ | 58.2 | North America | USA | Urban | English | Very High | QI | CIDI | 55 | 148 | 1817 | 8.1 |
| 3 | Hailemariam | 2012 | 36.1 | 51.6 | Africa | Ethiopia | Mixed | Other | Low | QI | ICD-10 | 98.7 | 449 | 4925 | 9.1 |
| 4 | Lindeman | 2000 | 15-75 | 55.8 | Europe | Finland | Mixed | Other | Very High | QI | CIDI-UM | 86 | 557 | 5993 | 9.3 |
| 5 | Ma | 2008 | 15-64 | 54.1 | Asia | China | Mixed | Other | High | QI | CIDI | 76.3 | 155 | 4767 | 3.3 |
| 6 | Mojtabai | 2004 | 50+ | 55.7 | North America | USA | Mixed | English | Very High | QI | CIDI-SF | 92.1 | 643 | 9747 | 6.6 |
| 7 | Newman | 1994 | 18+ | 61.7 | North America | Canada | Mixed | English | Very High | QI | DIS | 77.6 | 160 | 3070 | 5.2 |
| 8 | Peltzer | 2013 | 50+ | 42.7 | Africa | South Africa | Mixed | Other | Medium | QI | ICD-10 | 77 | 160 | 3840 | 4.2 |
| 9 | Wang | 2004 | 12+ | 53.5 | North America | Canada | Mixed | English | Very High | QI | CIDI-SF | 100 | 668 | 17244 | 3.9 |

MINI: Mini International Neuropsychiatric Interview; BDI: Beck Depression Inventory; PHQ: Patient Health Questionnaire; CIDI: Composite International Diagnostic Interview; EST-Q: Emotional state questionnaire; SCI DSM IV: Structured Clinical Interview DSM IV; HADS: Hospital Anxiety and Depression Scale; SPIKE: Structured Psychopathological Interview and Rating of the Social Consequences of Psychological Disturbances for Epidemiology; DASS-21: Depression Anxiety Stress Scales 21; GHQ-28: General Health Questionnaire; CIS-R: Clinical Interview Schedule-Revised; CES-D: Center for Epidemiologic Studies Depression Scale; PRIME-MD: Primary Care Evaluation of Mental Disorders screening questionnaire for depressive symptoms; SCAN: Schedules for Clinical Assessment in Neuropsychiatry; DIS: Diagnostic Interview Schedule; MDI: Major Depression Inventory; SCID-CV: Structured Clinical Interview for DSM Disorders; SDS: Self-rating Depression Scale.

**Supplementary Table 3** Characteristics of studies on lifetime prevalence of depression

| **No.** | **First author** | **Publication year** | **Age (mean years)** | **Female percent (%)** | **Continent** | **Country** | **Setting** | **Language** | **HDI** | **Screening method** | **Screening tool** | **Response rate (%)** | **Event** | **Sample size** | **Prevalence (%)** |
| --- | --- | --- | --- | --- | --- | --- | --- | --- | --- | --- | --- | --- | --- | --- | --- |
| 1 | Altamura | 1995 | 18+ | 51.9 | Europe | Italy | Mixed | Other | Very High | QI | CIDI | 86.9 | 33 | 480 | 6.9 |
| 2 | Angst | 1995 | 34-35 | 51.0 | Europe | Switzerland | Mixed | Other | Very High | QI | SPIKE | 68.9 | 106 | 407 | 17.9 |
| 3 | Beals | 2005 | 15-54 | 54.5 | North America | USA | Rural | English | Very High | QI | CIDI-UM | 74.3 | 167 | 3041 | 5.5 |
| 4 | Blazer | 1994 | 15-54 | 50.5 | North America | USA | Mixed | English | Very High | QI | CIDI | 82.4 | 1385 | 8098 | 17.1 |
| 5 | Carta | 1995 | 18+ | 52.1 | Europe | Italy | Mixed | Other | Very High | QI | CIDI | 87 | 64 | 480 | 13.3 |
| 6 | Chang | 2008 | 34.85 | 49.8 | Asia | South Korea | Mixed | Other | Very High | QI | CIDI | 69.1 | 223 | 5439 | 4.1 |
| 7 | Ialongo | 2004 | 19-22 | 55.9 | North America | USA | Urban | English | Very High | QI | CIDI-UM | 79.1 | 113 | 1197 | 9.4 |
| 8 | Kessler | 1997 | 15-54 | - | North America | USA | Mixed | English | Very High | QI | CIDI | 82.4 | 1054 | 6673 | 15.8 |
| 9 | Kessler | 2003 | 18+ | 44.5 | North America | USA | Mixed | English | Very High | QI | CIDI | 73 | 1472 | 9090 | 16.2 |
| 10 | Orui | 2011 | 54 | 52.5 | Asia | Japan | Rural | Other | Very High | QI | CIDI | 50.8 | 34 | 770 | 4.4 |
| 11 | Pakriev | 2009 | 18-59 | 54.5 | Europe | Russia | Urban | Other | High | QI | Russian MINI | - | 97 | 323 | 30.0 |
| 12 | Smith | 2013 | - | - | Europe | UK | Mixed | English | Very High | QI | CIDI | - | 7927 | 123000 | 6.4 |
| 13 | Tomlinson | 2009 | - | 53.7 | Africa | South Africa | Mixed | English | Medium | QI | CIDI | - | 422 | 4351 | 9.7 |

MINI: Mini International Neuropsychiatric Interview; BDI: Beck Depression Inventory; PHQ: Patient Health Questionnaire; CIDI: Composite International Diagnostic Interview; EST-Q: Emotional state questionnaire; SCI DSM IV: Structured Clinical Interview DSM IV; HADS: Hospital Anxiety and Depression Scale; SPIKE: Structured Psychopathological Interview and Rating of the Social Consequences of Psychological Disturbances for Epidemiology; DASS-21: Depression Anxiety Stress Scales 21; GHQ-28: General Health Questionnaire; CIS-R: Clinical Interview Schedule-Revised; CES-D: Center for Epidemiologic Studies Depression Scale; PRIME-MD: Primary Care Evaluation of Mental Disorders screening questionnaire for depressive symptoms; SCAN: Schedules for Clinical Assessment in Neuropsychiatry; DIS: Diagnostic Interview Schedule; MDI: Major Depression Inventory; SCID-CV: Structured Clinical Interview for DSM Disorders; SDS: Self-rating Depression Scale.
